# The effect of the National Health Insurance Scheme (NHIS) on health service delivery in mission facilities in Ghana: a retrospective study

**DOI:** 10.1186/s12992-016-0171-y

**Published:** 2016-06-07

**Authors:** Genevieve Cecilia Aryeetey, Justice Nonvignon, Caroline Amissah, Gilbert Buckle, Moses Aikins

**Affiliations:** University of Ghana, College of Health Science, School of Public Health, P. O. Box LG 13, Legon, Ghana; Christian Health Association of Ghana (CHAG), 21 Jubilee Street, Labone, P. O. Box AN 7316, Accra Ghana

**Keywords:** Health insurance, Mission facilities, Service delivery, Ghana

## Abstract

**Background:**

In 2004, Ghana began implementation of a National Health Insurance Scheme (NHIS) to minimize out-of-pocket expenditure at the point of use of service. The implementation of the scheme was accompanied by increased access and use of health care services. Evidence suggests most health facilities are faced with management challenges in the delivery of services. The study aimed to assess the effect of the introduction of the NHIS on health service delivery in mission health facilities in Ghana. We conceptualised the effect of NHIS on facilities using service delivery indicators such as outpatient and inpatient turn out, estimation of general service readiness, revenue and expenditure, claims processing and availability of essential medicines. We collected data from 38 mission facilities, grouped into the three ecological zones; southern, middle and northern. Structured questionnaires and exit interviews were used to collect data for the periods 2003 and 2010. The data was analysed in SPSS and MS Excel.

**Results:**

The facilities displayed high readiness to deliver services. There were significant increases in outpatient and inpatient attendance, revenue, expenditure and improved access to medicines. Generally, facilities reported increased readiness to deliver services. However, challenging issues around high rates of non-reimbursement of NHIS claims due to errors in claims processing, lack of feedback regarding errors, and lack of clarity on claims reporting procedures were reported.

**Conclusion:**

The implementation of the NHIS saw improvement and expansion of services resulting in benefits to the facilities as well as constraints. The constraints could be minimized if claims processing is improved at the facility level and delays in reimbursements also reduced.

## Background

Ghana is among the first countries in sub-Saharan Africa to begin implementation of a National Health Insurance Scheme (NHIS). Until the NHIS was introduced in 2003, the country had over time implemented a number of financing reforms. These reforms - with accompanying exemption policies - included general tax revenues and user fees [1] with the latter dominating the health financing scene from the early 1970s until 2003 when a National Health Insurance law was passed. Subsequently in 2004, Ghana begun implementation of the NHIS as a policy objective to minimize out-of-pocket expenditure at the point of use of service [2], thus reducing the financial barrier to health service utilization. The implementation of the scheme saw the registration of over 45 % of the Ghanaian population into the scheme by 2011 [3].

Since the introduction of the NHIS, studies have focused on assessing client interaction with the scheme as consumers, particularly on utilization and access to services [4–8], equity [9–11], client perceptions of quality of care [12, 13], client moral hazard behaviour among others [14, 15]. There is, however limited evidence on how providers are coping and managing the increased demand for health care services. For example, utilization and access to health services especially among the poor has increased [3] while most health facilities have not had corresponding increases in human and other resources to meet the increased demand. The NHIS requires facilities to provide documentation in processing and submission of claims by health care providers and this places a toll on already limited resources (i.e. time and personnel) in the facilities. Claims processing is an important component in the implementation of the scheme since providers could face losses in revenue should there be any rejections in claims submitted [16]. Again the timing and frequency of claims reimbursements has implications on the overall management of facilities.

Mission facilities are the second largest provider of healthcare in the country. They are identified as the Christian Health Association of Ghana (CHAG) member institutions. This organization is quasi-government primarily owned by 21 Christian religious organisations. CHAG currently has 183 health facilities and training institutions providing care for the most vulnerable and underprivileged population groups in all 10 Regions of Ghana, particularly in the most remote areas. CHAG is autonomous and takes an independent position to advocate and promote improvements in the health sector and to promote the interest of its members and its target beneficiaries. By 2010, 90 of the facilities had received accreditation to provide care for clients of the NHIS. Again, some of these facilities have experimented with health insurance, in the form of community based health insurance schemes prior to the introduction of the NHIS in 2003 [17], giving these facilities some foreknowledge of operations of health insurance. CHAG’s experiences in operating health insurance schemes in some of their facilities within their communities provided information towards implementation of the current health insurance scheme in Ghana [18].

Against this background, this study set out to assess the effect of the introduction of health insurance on service delivery of mission facilities in Ghana. The study‘s value is in the provision of evidence on how providers respond to the challenges that come with implementation of the scheme.

## Methods

### Study design

A retrospective cross-sectional study was undertaken in November 2012, collecting information from two time periods-2003 and 2010.

### Selection of study facilities

The study facilities were selected from the 183 CHAG member institutions operating under various capacities as Specialist centres (3), hospitals (59), clinics (77), primary health centres (15), and health centres (19); training institutions (10) were not included in this study. Our sampling frame was the 90 facilities that had received accreditation by 2010. We selected all the three specialist centres, sampled hospitals, clinics, health centres and PHC centres by probability proportional to size based on the number and type of facility per region. Further, the regions were categorized into the three ecological zones, namely, savannah (northern), forest (middle) and coastal (southern). In total, five facilities in the northern, 17 in the middle and 12 in the southern zones making a total of 34 facilities were selected (Table 1).

**Table 1:** Facilities and key health services provided

	Zone	Name of facility	Type of facility	Key health services provided
1	Middle	Methodist Faith Healing Hospital, Ankaase	Hospital	Outpatient/inpatient services
2	Middle	Seventh Day Adventist Hospital, Asamang	Hospital	Outpatient/inpatient services
3	Middle	Akoma Memorial SDA Hospital, Kumasi	Hospital	Outpatient/inpatient services
4	Middle	Methodist clinic, Bosomtwi	Clinic	Outpatient services
5	Middle	Aburaso Methodist Clinic, Atwima Kwakuma	Clinic	Outpatient services
6	Middle	Church of Christ mission clinic	Clinic	Outpatient services
7	Middle	Anglican eye clinic	Specialist Centre	Eye/Ophthalmic services
8	Middle	Benito Menni health centre	Health Centre	Outpatient services
9	Middle	Sacred heart health centre	Health Centre	Outpatient services
10	Middle	Holy family Hospital	Hospital	Outpatient/inpatient services
11	Middle	Presbyterian hospital, Brong Ahafo	Hospital	Outpatient/inpatient services
12	Middle	St John of God	Specialist Centre	Orthopaedics
13	Middle	Janie speaks A.M.E Zion	Health Centre	Outpatient services
14	Middle	Presbyterian Health Centre, Abetifi	Health Centre	Outpatient services
15	Middle	St. Martin's de Porres Hospital	Hospital	Outpatient/inpatient services
16	Middle	Abetifi presby health centre	Health Centre	Outpatient services
17	Middle	Prebyterian clinic	Clinic	Outpatient services
18	Northern	Baptist Medical Centre, Nalerigu	Hospital	Outpatient/inpatient services
19	Northern	Catholic PHC, Bole	Primary Health Centre	Outpatient services
20	Northern	St. Theresa Health Centre, Zorko	Health Centre	Outpatient services
21	Northern	Martyrs of Uganda Health Centre	Health Centre	Outpatient services
22	Northern	Presbyterian PHC, Bolgatanga	Primary Health Centre	Outpatient services
23	Southern	Presbyterian clinic, Assin South	Clinic	Outpatient services
24	Southern	St. Gregory Catholic clinic	Clinic	Outpatient services
25	Southern	Manna Mission Hospital, Teshie-Nungua	Hospital	Outpatient/inpatient services
26	Southern	Emmanuel Eye Centre	Specialist Centre	Eye/Ophthalmic services
27	Southern	St. Andrew's Clinic and Maternity	Clinic	Outpatient services, antenatal
28	Southern	Mary Theresa Hospital, Dodi-Papase	Hospital	Outpatient/inpatient services
29	Southern	The Salvation Army Clinic, Adaklu Sofa	Clinic	Outpatient services
30	Southern	Matter Ecclesiae, Sokode	Clinic	Outpatient services
31	Southern	St. Martin De Pores hospital	Hospital	Outpatient/inpatient services
32	Southern	Holy child Clinic, Sekondi	Clinic	Outpatient services
33	Southern	Pentecost Clinic	Clinic	Outpatient services
34	Southern	Holy child Clinic, Ahanta	Clinic	Outpatient services

### Data collection

Annual data were collected for time periods, 2003 and 2010, the former being the immediate year preceding the introduction of health insurance and the latter being five years after the introduction of the scheme (a period long enough to observe any effects on service delivery) but also the first year after introduction for which complete data were available as at the time of data collection. Further, providers would have also gained familiarity with the scheme to make suggestions for improvement.

Data were collected on various aspects of service delivery. Annual reports of facilities were reviewed for information on resources used, financial data and selected service delivery indicators, mainly outpatients and inpatient visits. Data on facility revenue and expenditures were also collected. Data on indicators for service readiness such as basic amenities, basic equipment, standard precautions for prevention of infections, laboratory equipment, and availability of essential medicines were also collected.

### Data analysis

We analysed different aspects we considered related to delivery of services by health facilities. First, outpatient attendance for all facilities and inpatient attendance for hospitals only. Second, we estimated facilities’ overall service readiness using a general service readiness (GSR) indicator developed by WHO. This indicator identifies five (5) domains to which facilities can be assessed for readiness to offer services. They include infrastructure, basic supplies, standard precautions, laboratory tests, medicines and commodities. A facility’s readiness to offer service is based on a cumulative score from the five domains and ranges from 0 to 1 with higher values (>0.76) representing readiness to deliver services [19].

Other issues related to service delivery that were analysed were revenue and expenditure, claims submission/reimbursements and availability of essential medicines. We estimated total expenditures and internally generated funds before and after the NHIS. These indicators were analysed using a before-after approach.

For claims, we estimated patterns of claims reimbursements, errors in claims submission and percentage claims not reimbursed after the NHIS. The Ghanaian currency figures were converted to US dollar equivalent using mid-year exchange rate for 2003 and 2010 [20]. Availability of essential medicines was also analysed based on a list of common disease conditions reported at facilities. Data analysis outputs are presented in tables and charts.

### Ethical approval and consent

With regard to ethical approval, no formal approval was sought from any Institutional Review Board (IRB) in Ghana for this study. This was because researchers from the School of Public Health (SPH), University of Ghana, were contracted by CHAG to undertake this study. CHAG is the governing body of mission health facilities in Ghana. Management of selected study mission health facilities were directly informed of the study by CHAG given that these facilities were under their control. SPH in conjunction with CHAG designed the study and SPH did the data collection and analyses. Verbal consent were obtained from selected clients at these facilities before interviews.

## Results

### Outpatient and inpatient visits

The total outpatient and inpatient visits for all facilities before introduction of the NHIS (i.e. 2003) were 1,677,731 and 141,243 respectively, and 2,749,405 outpatient visits and 213,175 inpatient visits after NHIS (i.e. 2010). This represented an increase of 64 % and 51 % respectively over the period. Whereas hospitals in the southern and northern zones recorded increases in outpatient visits of 56 % and 29 % respectively, hospitals in the middle zone recorded 1 % increase after the introduction of the NHIS. With respect to inpatient visits, hospitals in the southern and middle zones recorded increases of 48 % and 63 % respectively whilst hospitals in the northern zone recorded a marginal increase of about 0.1 % (Table 2). The largest increases in outpatient visits were recorded by specialist centres in the middle zone followed by PHCs in the northern zone.

**Table 2:** Distribution of outpatient and inpatient attendance in CHAG facilities before and after NHIS

Ecological zones	Type of facility	Total OPD visits before NHIS	Total OPD visits after NHIS	% increase (after NHIS)	Total inpatient visits before NHIS	Total inpatient visits before NHIS	% increase (after NHIS)
Southern	Hospital	495,035	770,667	56	69,552	102,620	48
	Clinic	61,056	292,183	3.79 times^a^	-	-	
	Health Centre	4,276	18,733	3.38 times^a^	-	-	
	Specialist Centre	29,568	30,960	5	-	-	
Middle	Hospital	699,082	707,089	1	62,100	100,958	63
	Clinic	296,229	713,165	1.41 times^a^	-	-	
	Health Centre	15,794	58,675	2.72 times^a^	-	-	
	PHC	-	4,833	-	-	-	
	Specialist Centre	1,384	16,373	10.83 times^a^	-	-	
Northern	Hospital	73,506	94,828	29	9,591	9,597	0.06
	Health Centre	-	25,837	-	-	-	
	PHC	1,800	16,062	7.92 times^a^	-	-	
Total	Hospital	1,267,624	1,572,584	24	141,243	213,175	51
	Clinic	357,285	1,005,348	1.81 times^a^	-	-	-
	Health Centre	20,070	103,245	4.14 times^a^	-	-	-
	PHC	1,800	20,895	10.61 times^a^	-	-	-
	Specialist Centre	30,952	47,333	53	-	-	-
	Total	1,677,731	2,749,405	64	141,243	213,175	51

^a^The increase was over 100 %

### General Service Readiness (GSR)

**Fig. 1:**
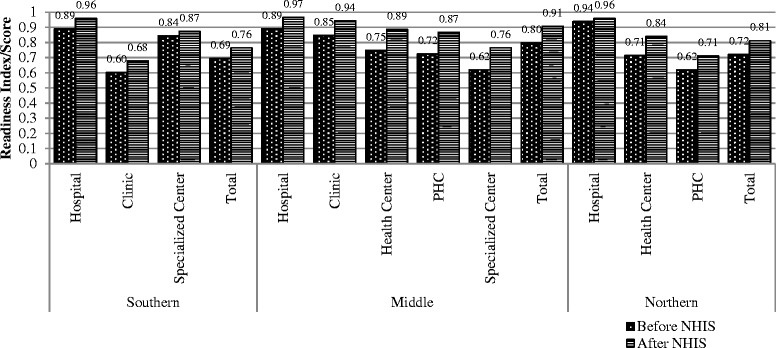
General services readiness scores

Figure 1 presents the general services readiness scores (GSR) categorized by type of facility and zone. The figure shows overall improvement in GSR from 2002 to 2010 as follows; in the southern zone reported an increase from 0.69 to 0.76, the middle zone from 0.80 to 0.91 and the northern from 0. To 0.81. Facilities that recorded lowest GSR scores in 2003 were clinics (0.60) in the southern zone, specialist centers (0.62) in the middle zones and PHCs (0.62) in the northern zones. Interestingly these facilities recorded the lowest scores also in 2010.

### Expenditure and internally generated funds

The total annual expenditure for all facilities increased from US$4,748,385.10 before the introduction of NHIS to US$66,867,518.22 after NHIS while internally-generated funds increased from US$4,947,569.97 to US$41,505,083.73 accordingly (Table 3). The largest increases in both expenditure and IGF were recorded by specialist centres, from US$20,825.40 to US$11,383,097.20 and from US$10,708.07 to US$1,139,535.91 respectively.

**Table 3:** Expenditure and IGF before and after NHIS (US$)

Type of facility	Total expenditure before NHIS	Total expenditure after NHIS	Total IGF before NHIS	Total IGF after NHIS
Hospital	4,417,146.27	41,022,555.86	4,324,357.71	34,005,075.07
Clinic	141,084.94	10,413,869.27	507,991.82	4,973,039.68
Health Centre	134,412.60	3,421,049.38	104,512.36	773,006.77
PHC	34,915.89	626,946.50	-	614,426.30
Specialist Centre	20,825.40	11,383,097.20	10,708.07	1,139,535.91
Total	4,748,385.10	66,867,518.22	4,947,569.97	41,505,083.73

**Fig. 2:**
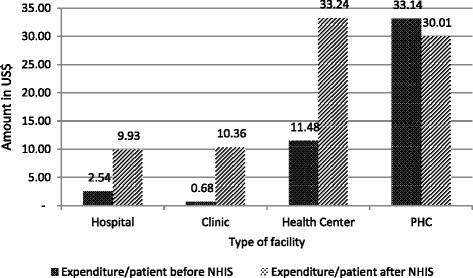
Expenditure per patient before and after NHIS

In addition, expenditure per patient (out-patient services) increased for all types of facilities^1^ (except PHCs) after the NHIS compared to before the NHIS. Hospital expenditure per patient increased by about four times while those for clinics and health centres were about 15 and 3 times respectively. For in-patient services, cost per patient day equivalent for hospitals was calculated^2^ to be US$ 1.02 before the NHIS and US$3.67 after the NHIS (Fig. 2).

### Claims submission and reimbursement

On average, the value of claims submitted by hospitals in the southern zone to NHIA was US$859,964.76 with reimbursement value of US$ 839,122.44 indicating about 2.4 % of claims submitted not reimbursed. The case of hospitals in the northern zone was no different - about 1 % of the claims submitted was not reimbursed. The percentage of claims submitted by hospitals in the middle zone which was not reimbursed was 13 %, higher than hospitals in southern and northern zones. In addition, within the southern zone, clinics had the highest proportion of claims that were not reimbursed (46 %) whilst specialist centres had the lowest non-reimbursement rate of less than 1 %. A similar trend was recorded for facilities in the middle zone where 48 % of claims submitted by clinics were not reimbursed. In the northern zone, health centres recorded the highest non-reimbursement rate (about 13 %), whilst PHCs recorded the lowest non-reimbursement rate (1 %).

**Fig. 3:**
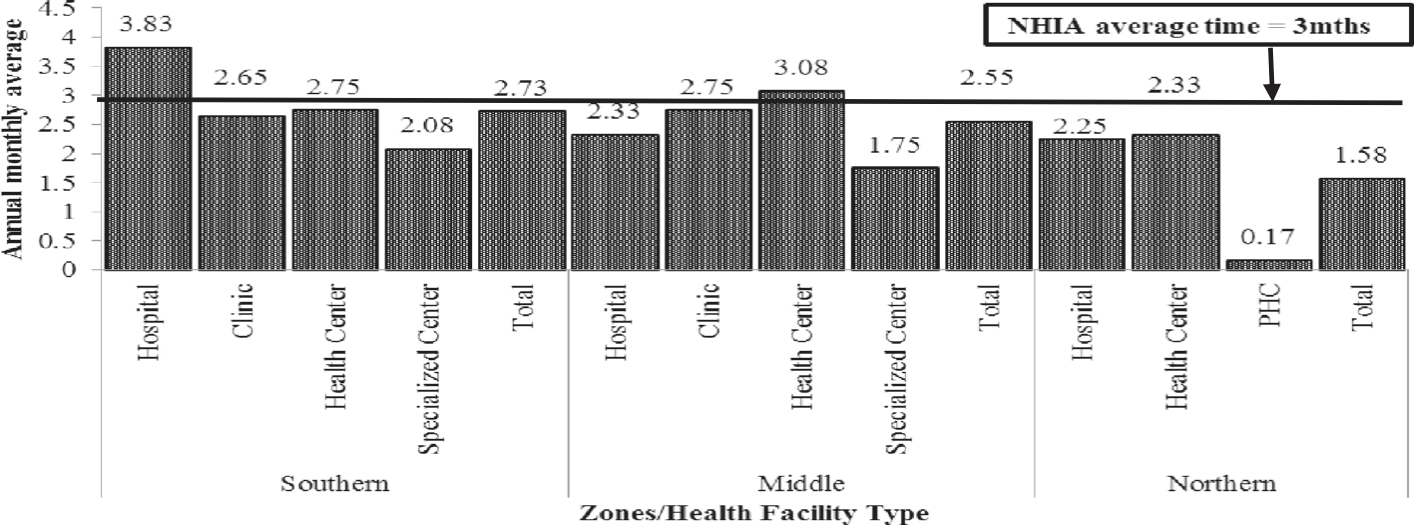
Period between claims submission and reimbursement, 2010

The reimbursement of NHIS claims by the NHIA to facilities takes between 1 to 4 months after submission of claims. More specifically, hospitals in the southern zone and health centres in the middle zone had to wait for between 3 to 4 months to be reimbursed whilst PHCs in the northern zone wait for less than 1 month after claims submission, on average (Table 4, Fig. 3).

**Table 4:** Average annual NHIS claims submissions and reimbursements, 2010^a^

Ecological Zone	Type of facility	Claims submitted (US$^b^)	Claims reimbursed (US$)	% claims not reimbursed	Number of facilities
Southern	Hospital	859,964.76	839,122.44	2	3
	Clinic	360,472.47	193,086.49	46	8
	Health Centre	133,442.80	122,804.05	8	1
	Specialist Centre	68,185.79	67,742.28	1	1
Middle	Hospital	960,733.77	838,349.19	13	5
	Clinic	199,697.26	103,593.36	48	4
	Health Centre	102,319.02	97,282.30	5	6
	Specialist Centre	487,763.16	399,354.13	18	2
Northern	Hospital	218,340.38	215,503.77	1	1
	Health Centre	71,572.64	62,609.66	13	2
	PHC	29,612.36	29,266.98	1	2

^a^The table represents facilities for which NHIS claims data were readily available; ^b^US$ and Ghana Cedi exchange rate was GHC1.4187 to US$1 in 2010

### Availability/non-availability of essential medicines

Table 5 presents the non-availability of selected essential medicines in facilities (excluding specialist centres) before and after introduction of the NHIS. The table revealed that there was a general improvement in availability of essential medicines in facilities in all the three ecological zones after the introduction of the NHIS. In the Northern zone however, the percentage of essential medicines not available remained the same for indicators (conditions) such as diabetes, cardiovascular diseases and depression while increasing for conditions related to the central nervous system and ulcers (i.e. from 25 to 50 %).

**Table 5:** Non-availability of essential medicines (%)

		Southern zone		Middle zone		Northern zone	
Indication	Medicine name	Before	After	Before	After	Before	After
Malaria	ACTs	53.80	7.70	33.30	0	40.00	0
Asthma	Salbutamol	25.00	7.70	26.70	6.20	0	20.00
Diabetes	Gilbendamide	38.50	30.80	20.00	18.80	60.00	60.00
Cardiovascular disease	Atenolol	58.30	23.10	20.00	25.00	40.00	40.00
Depression	Amitriptyline	58.30	38.50	33.30	50.00	60.00	60.00
Infectious disease	Ciprofloxacin	8.30	0	13.30	6.20	20.00	0
Infectious disease	Co-trimoxazole	25.00	7.70	6.00	6.20	0	0
Infectious disease	Amoxiillin	15.40	7.70	13.30	6.20	20.00	0
Central nervous system disease	Diazepam	15.40	7.70	13.30	6.20	20.00	50.00
Ulcer	Omeprazole	30.80	15.40	26.50	18.80	25.00	50.00

## Discussion

Our study has revealed that on the whole, there were general improvements in service delivery after the introduction of the NHIS. This notwithstanding, the facilities also enumerated significant challenges that need to be addressed to facilitate improvement in the delivery of health services to their catchment populations.

The results of the study show that outpatient visits increased by 64 % and inpatient visits increased by 51 %. These results indicate high service utilization as a result of the introduction of the NHIS. Indeed many studies in Ghana and other developing countries have shown that the implementation of a health insurance scheme (national or community based) results in people seeking for formal care once insured [8, 21, 22]. The Ministry of Health (MOH) in their 2013 annual report explained that the increases in utilization can be attributed to increase in outpatient attendance by the insured [23]. Again, Yawson et al. reported mean out-patient attendance to be between 1.0-2.48 by the insured and 0.39-1.18 by the uninsured [14]. This increase in outpatient attendance could imply increased revenue to facilities, at the same time increased burden on health infrastructure which must see proportionate improvements to cope with the increased workload. Facilities thus need to set aside portions of their revenue to undertake infrastructural expansions so as not to compromise the quality of their outputs i.e. services delivered.

Furthermore, the results show that the general service readiness of all facilities improved after the introduction of the NHIS. It is worth noting that specialist centres in the middle zone, had the largest increase in outpatient visits and recorded the highest improvement in service readiness. This may be attributed to improved infrastructure and other equipment but may also be due to improved efficiency of operations. The effect of service readiness can be interpreted in patient’s perceptions of quality of care and patients’ satisfaction with quality of care after introduction of the health insurance scheme in the country. Studies have shown that generally, quality of care has improved for both the insured and uninsured even though sometimes insured patients have to wait for longer hours to be attended to [24, 25].

The findings also show that facilities’ spending per patient increased after the introduction of the NHIS compared to the period before the NHIS. In economic terms, this denotes inefficiency in spending. Plausible reasons for this include the extra spending that facilities make on NHIS members that is not reimbursed by the NHIA. A notable example – which also came out of the in depth interviews with administrators is the expenditure on documentation. The reimbursements do not cover documentation and facilities may also have made expenditure on other items (e.g. building) which do not necessarily relate directly to the NHIS.

With regard to health expenditure, the results showed that overall, there was a nine-fold increase in expenditure of all facilities and a seven-fold increase in internally-generated funds. If the facilities invested more in infrastructure, the high increases in expenditure could be justified as improved infrastructure produces long term benefits. However, if these increases were as a result of other activities that do not yield better returns, then the situation could be worrying. Facilities need to evaluate their spending patterns to ensure that they do not reflect inefficiencies in management. It is important to note that the large increases in outpatient visits at specialist centres reflects the large increase in IGF, and similarly large increase in expenditure and (possibly) investment in critical infrastructure.

The total claims reimbursed to facilities represented about 59 % of the total expenditure of these facilities in 2010. Thus, revenue generated from NHIS alone did not seem to match facilities’ expenditure in the short-run. Admittedly, facilities have other sources of revenue which may compensate for the shortfalls. Moreover, some of the expenditures were likely to be investment (i.e., capital items which have longer useful lives) that may have long term benefits. The results further revealed high rate of non-reimbursement of NHIS claims, particularly among clinics in the middle and southern zones and low rates of non-reimbursement among PHCs. Among the reasons cited for the high non-reimbursement rates were errors in claims processing, no feedback from NHIA regarding the nature of errors and lack of clarity on reporting procedures. The plausible reasons for the marked differences in non-reimbursement between facility types are that PHCs generally have lower utilization (workload) compared to clinics; thus, more time for appropriate staff to spend on managing claims. This was observed during interaction with accounting and managerial staff. The fairly low rates of non-reimbursement among hospitals – particularly in southern and middle zones – may be attributed to the comparatively higher calibre of staff who are dedicated to managing claims at these higher level facilities. Anecdotal evidence and some studies have reported that these errors results in long delays in claims reimbursements, which this study as reported to be around 3 months [26]. Nonetheless, facility administrators must put in place administrative structures to reduce financial losses due to errors in claims processing.

The availability of essential medicines in the right quality and price are core in service delivery. The study showed that all facilities improved the availability of essential medicines after the introduction of the NHIS compared to the period before the NHIS. Similar studies in Ghana have found that in as much as utilization and availability of medicines have increased, there were low reimbursement rates for medicines which result in providers asking patients to pay supplementary fees. Sometimes, medicine supplies were also intermittent [27, 28]. Thus there is room for further improvements especially in facilities with limited access to medicines, for our study this will be in the northern zones.

A number of issues are important in the interpretation of our results. First we recognise competing interventions at around the time of implementation of the NHIS which include WHO’s universal health coverage agenda, the Millennium Development Goals (MDGs), with target on health and other local policies. These interventions influence health service delivery in one way or the other and thus we cannot associate all outcomes in our results to be effect of the NHIS, though in the case of the MDGs, the implementation of interventions towards meeting the MDGs begun shortly after their adoption and before the introduction of the NHIS in 2004. Thus, it could be argued that the effects of the MDGs may have been the same before and after the introduction of the NHIS. Notwithstanding, the MDGs gathered momentum with time, and it is expected that a lot more was done after 2004. Second, this study collected data from two time points, one before and one after. A time trend analysis for different years before and after would have been more appropriate. However, shortfalls in data availability imposed a limit on what analysis could be done, limiting the study to the two time points.

It is important to note that service delivery is only one of the components of the health system, with the other components being leadership and governance, health workforce, medical products and technologies, health information, and health financing. Thus, given the interconnectedness of the systems building blocks, any effects of the NHIS (i.e. financing) on service delivery would lead affect other building blocks of the health system [29]. For simplicity, however, the current study focuses on service delivery, though it is acknowledged that the effects of financing on service delivery would be observed in access, safety and quality and ultimately in efficiency, responsiveness and equity in health.

## Conclusion

In general terms, with the introduction of the NHIS, mission facilities improved and expanded their services to meet the increased utilization. This brought in its wake financial and other materials benefits to the facilities. Nonetheless, errors in claims leading to large non-reimbursements if unchecked could mar the benefits derived from NHIS. We recommend that mission facilities engage with the health insurance authority (NHIA) to provide clear feedback on the nature of the errors and find ways to minimize these errors. The authority should also conduct periodic training of facilities on claims processing. It is recommended that facilities may hire staff specifically to take care of claims processing to ease the burden on health workers to focus on their core duties. Finally given the increased utilization and in order not to sacrifice quality of care, facilities should invest in expanding infrastructure to meet the increased demand.

## Abbreviations

CHAG, Christian Health Association of Ghana; GSR, General Service Readiness; IRG, Institutional Review Board; MOH, Ministry of Health; NHIA, National Health Insurance Authority; NHIS, National Health Insurance Scheme; PHC, Primary Health Centers; SPH, School of Public Health; WHO, World Health Organization.

## Footnotes

1 The figure for specialist facilities was considered as an outlier and was, therefore, dropped from the graph.

2 Based on the assumption that a hospital stay is equivalent to 3 OPD visits and average length of stay is 4 days, hence hospital stay is weighted as equivalent to 12 OPD visits (Mbananga et al. [30]). This assumption was used to extrapolate inpatient expenditure. Total hospital days were estimated as 4 times total admissions.
